# Regulation of Chemokine–Receptor Interactions and Functions

**DOI:** 10.3390/ijms18112415

**Published:** 2017-11-14

**Authors:** Martin J. Stone

**Affiliations:** Infection and Immunity Program, Monash Biomedicine Discovery Institute and Department of Biochemistry and Molecular Biology, Monash University, Clayton VIC 3800, Australia; martin.stone@monash.edu; Tel.: +61-3-9902-9246

Inflammation is the body’s response to injury or infection. As early as 2000 years ago, the Roman encyclopaedist Aulus Cornelius Celsus recognised four cardinal signs of this response—redness, heat, swelling and pain; a fifth sign is loss of function. The underlying cause of these common symptoms remained a mystery until the 19th century, when Rudolf Virchow “claim(ed) for the leukocyte a place in the field of pathology” [1]. It is now widely recognised that all inflammatory responses involve migration of leukocytes (white blood cells) to the affected tissues, where they accumulate and carry out a plethora of functions including elimination of pathogens, regulation of immunity and tissue repair.

While leukocyte recruitment is a beneficial response to pathogen invasion, we are all too familiar with the detrimental roles it can play in numerous diseases. As an example, in allergic asthma, the recruited leukocytes include eosinophils, which can then undergo degranulation, releasing toxic proteins that induce airway constriction and difficulty breathing [2]. In this case, the inflammatory response causes more damage than the initial stimulus (the allergen), so it would be beneficial to suppress the leukocyte recruitment. The same is true in many other inflammatory diseases, such as atherosclerosis, rheumatoid arthritis, multiple sclerosis, dermatitis, etc. However, it is essential that such a therapeutic strategy should not suppress inflammation so much as to make the patient susceptible to infections. To successfully achieve the right balance between the “yin and yang” of inflammation, we need to understand the biochemical mechanisms underlying leukocyte recruitment.

Enter chemokines and chemokine receptors. Over a period of about 20 years beginning in the late 1980s, researchers discovered a family of related proteins that are secreted by various cell types as an early response to tissue damage and attract leukocytes towards the affected tissues. These proteins were named chemokines (chemotactic cytokines) due to their ability to induce chemotaxis, which is migration of cells towards a chemical stimulus. They elicit this function by activating chemokine receptors, a family of G protein-coupled receptors expressed on the surfaces of leukocytes. Importantly, different types of leukocytes express different chemokine receptors, so much of the selectivity of leukocyte responses, i.e., which types of leukocytes are recruited in a given situation, arises from the complementarity between the specific chemokines expressed in the affected tissues and the specific receptors expressed on the different leukocytes. The chemokine–receptor network was clearly an attractive target to suppress unwanted inflammation while still enabling appropriate responses to pathogens.

Following the discovery of chemokines and their receptors, there has been an enormous body of research exploring their roles in normal physiology and inflammatory diseases as well as the mechanistic basis of their activity. Indeed, a PubMed search for “chemokine” now gives almost 150,000 hits. Pick an inflammatory disease of interest and there is a good chance you will be able to find analyses of affected tissues showing the types of leukocytes recruited and elevated concentrations of the chemokines responsible. In many cases, knockout mice or pharmacological inhibition studies have shown that eliminating the relevant chemokine–receptor interactions significantly reduces inflammatory symptoms. These results have emboldened drug companies to develop small molecules or biologics that target chemokine receptors (or occasionally chemokines) and to test them in clinical trials against a wide range of inflammatory diseases [3].

However, despite much hope and the investment of billions of dollars, the results have been disappointing and most trials have failed. The reasons are complex and varied but commonly the tested drugs do not show the same efficacy or target selectivity in humans as they did in animal models. Perhaps this should not be surprising as the chemokine–receptor networks differ between species, and drugs that exhibit high specificity in one species could easily have off-target effects in another. Moreover, even in a single species, inhibition of one receptor may not be sufficient to block a response if alternative, compensatory receptors are still active.

Clearly, much of the difficulty in successfully targeting chemokines and their receptors arises from the complexity of their biology. Not only are these protein families extensive and promiscuous but there are numerous mechanisms known by which their activities are modulated. Despite a substantial body of basic research, our understanding of these mechanisms remains incomplete and more work is needed. Fortunately, chemokines and their receptors have caught the attention of many basic researchers who continue to explore their structures, biochemical functions, modes of interaction, pharmacology and mechanisms of regulation. This Special Issue highlights a variety of approaches being taken to elucidate these aspects of chemokine–receptor function.

As an introduction to this Special Issue, my colleagues and I have reviewed the variety of mechanisms by which chemokines and their receptors can be regulated [4], summarized schematically in Figure 1 of this review. We give an overview of the two protein families, and their network of selective interactions and we discuss what is known about the structural basis of these interactions. We highlight the variation of chemokine and receptor primary sequences through polymorphisms, mutations, splice variants and proteolytic modifications and we describe a variety of other post-translational modifications that can enhance or reduce their functions, either directly or by altering their stability or localization. In addition, we explore the complexity of downstream cellular signals stimulated by chemokines acting upon their receptors and give a brief overview of natural and synthetic inhibition approaches.

Our review also touches on the oligomerization of both chemokines and chemokine receptors and the interactions of chemokine oligomers with glycosaminoglycans (GAGs), which affects biological activity by enabling the formation of chemokine gradients that promote leukocyte chemotaxis. These latter two topics are discussed in more detail in two other review articles in this issue. Miller and Mayo provide an in-depth analysis of the tertiary and quaternary structures of chemokines, and their functional consequences, with a particular focus on the phenomenon of heterodimerization [5]. Considering that most chemokines homodimerize and the dimerization interfaces are largely conserved within each of the two major subfamilies of chemokines (CC and CXC), it makes sense that different chemokines from the same subfamily can form heterodimers with each other. This greatly increases the number of dimeric species that could be present within the biological milieu, binding to GAGs and swapping chemokine protomers with each other. As discussed by Thompson et al. [6], the situation is further complicated by the variety of different GAG structures, the influence of GAGs on chemokine oligomers and the effects of the tissue microenvironment.

The interplay between chemokine heterodimerzation and GAG binding is evident in the two articles by Brown et al. in this Special Issue [7,8]. First, they show that the chemokine CXCL7, which exists in equilibrium between monomeric and dimeric forms, is able to bind to GAGs even in its monomeric state [7], although structural modeling suggests that dissociation from GAGs is a prerequisite for receptor binding. Second, they demonstrate that CXCL7 heterodimerizes with several other CXC chemokines and they use a trapped heterodimer to show that the GAG interactions of the heterodimer are distinct from those of the CXCL7 monomer [8].

In addition to their heterogeneity of three-dimensional structure and GAG-binding, chemokines can also vary substantially in their covalent molecular structures due to heterogeneous post-translational modifications. One common modification is limited proteolysis, which commonly alters the functionally important N-terminal regions of chemokines. Illustrating this effect, Metzemaekers et al. show that three chemokine ligands of the receptor CXCR3 are all inactivated by N-terminal cleavage but that GAGs protect the chemokines from this cleavage while also competing with the receptor for chemokine binding [9]. Another post-translational modification of chemokines is the nitration of various amino acid side chain groups by reactive nitrogen species. In their review article, Thompson et al. [6] discuss this modification and its consequences for recognition of both chemokine receptors and GAGs. An additional modification, investigated by Nguyen et al. [10], is the cyclization of an N-terminal glutamine residue to yield pyroglutamate. Considering the importance of the chemokine N-terminus for function, this modification has the potential to influence receptor interactions. However, the authors show that pyroglutamate formation (and other N-terminal modifications) do not substantially affect the potency of 5P12-RANTES, a variant of RANTES/CCL5 that inhibits HIV entry via the chemokine receptor CCR5. They also define the kinetics of N-terminal cyclization, which may influence the functions of other chemokines such as the monocyte chemoattractant proteins (MCPs).

Just as chemokines may be post-translationally modified, so too can their receptors. One important modification is the sulfation of tyrosine residues in the N-terminal regions of the receptors, thought to be the initial site of chemokine interaction. A number of previous studies have demonstrated that tyrosine sulfation enhances chemokine binding affinity and, in some cases, can alter chemokine binding selectivity [11]. In this Special Issue, Moussouras et al. provide a new example of the latter effect [12]. They demonstrate the application of computational solvent mapping to identification of sulfotyrosine-binding hot spots on the surfaces of several CXC chemokines and experimentally validate their prediction that sulfotyrosine would bind specifically to some chemokines but not others.

Studies of tyrosine sulfation of chemokine receptors (and other proteins) have been challenging due to the difficulties generating sufficient quantities of homogeneously sulfated receptors or receptor fragments. In spite of recent progress in these methods [13], sulfated proteins and peptides will always suffer from marginal and variable stability. Therefore, it may be advantageous to use sulfotyrosine analogues with enhanced stability. To this end, Phillips et al. present a comparison of CCR7-derived peptides containing sulfotyrosine and the more stable analogue phosphotyrosine [14]. Importantly, they show that the phosphorylated peptides retain the same binding site specificity as the sulfated peptides, thus supporting their future utility as sulfopeptide surrogates.

It is well established that cells expressing chemokine receptors exhibit a variety of signaling responses to the cognate chemokine ligands of those receptors. In this Special Issue, Adamski et al. report a remarkable variation on this paradigm, showing that cells expressing the chemokine CXCL16 can also respond to the corresponding receptor CXCR6 [15]. This “reverse signaling” effect is only possible because the chemokine domain of CXCL16 is linked, via a long mucin stalk, to a transmembrane helix and a short cytoplasmic domain. The authors demonstrate that the reverse signaling is reliant on the cytoplasmic domain of CXCL16. Moreover, their finding that CXCL16 expression was increased in fast-migrating glioblastoma cells suggests that the observed reverse signaling may have important consequences for tumor cell migration (metastasis).

In searching for effective strategies to inhibit chemokines and their receptors, researchers have explored a wide variety of approaches. One approach is to use proteins naturally produced by pathogens to suppress chemokine-mediated inflammation during infection. To this end, Nguyen et al. describe their biophysical studies of two poxvirus proteins, one of which broadly inhibits mammalian chemokines while the other inhibits chemokine receptors [16]. They find that these two proteins bind extremely tightly to each other and propose a structural basis for the high affinity interaction. This study may help to guide the development of protein-based therapeutics but also raises questions about the balance between these proteins binding to each other versus inhibiting host inflammation during viral infection.

Although inhibition of chemokines or their receptors is an attractive strategy against inflammation and tumor metastasis, a confounding factor is that some chemokine–receptor interactions have important homeostatic or protective functions. An example is described by Sakumoto et al., who report that the expression levels of several chemokines and chemokine receptors are elevated in the endometrium of cows during pregnancy [17]. The increase in some of these proteins appears to be regulated by interferon τ, which acts as a bovine reproductive hormone, leading the authors to suggest that the chemokines and receptors may contribute to the maintenance of normal endometrial function during pregnancy.

The articles in this Special Issue emphasize the remarkable range of mechanisms by which the chemokine–receptor network is regulated in nature and can potentially be controlled therapeutically. The diversity of these mechanisms underlines the ongoing evolutionary battle between pathogens and their hosts and the subtle balance between beneficial and detrimental biological outcomes. While much remains to be learned, fundamental mechanistic studies, such as those described herein, will continue to provide invaluable guidance in the development of effective pharmaceutical interventions for many inflammatory diseases.
